# The role of extracorporeal CO_2_ removal from pathophysiology to clinical applications with focus on potential combination with RRT: an expert opinion document

**DOI:** 10.3389/fmed.2025.1651213

**Published:** 2025-09-01

**Authors:** Francisco José Parrilla-Gómez, Antonio Castelli, Riccardo Colombo, Antero do Vale-Fernandes, Federico Nalesso, David Pestaña-Lagunas, Fernando Suarez-Sipmann, Pierpaolo Terragni

**Affiliations:** ^1^Critical Care Department, Hospital del Mar, Barcelona, Spain; ^2^Department of Medicine and Life Sciences (MELIS), Hospital del Mar Research, Institute (IMIM), Critical Illness Research Group (GREPAC), Universidad Pompeu Fabra (UPF), Barcelona, Spain; ^3^Anesthesia and Intensive Care Unit, ASST Fatebenefratelli-Sacco, Luigi Sacco Hospital, Milan, Italy; ^4^Serviço de Medicina Intensiva, Unidade Local de Saúde Almada - Seixal (ULSAS), Hospital Garcia de Orta, Almada, Portugal; ^5^RISE-Health, Faculdade de Ciências da Saúde, Universidade da Beira Interior, Covilhã, Portugal; ^6^Faculdade de Ciências da Saúde, Universidade Fernando Pessoa, Porto, Portugal; ^7^Faculdade de Medicina da Universidade de Lisboa, Lisbon, Portugal; ^8^Nephrology, Dialysis and Transplantation Unit, Department of Medicine, University of Padova, Padova, Italy; ^9^Department of Anaesthesia and Intensive Care, Hospital Universitario Ramon y Cajal, IRYCIS, Madrid, Spain; ^10^Department of Health Science, School of Medicine, Universidad de Alcalá, Alcalá de Henares, Madrid, Spain; ^11^Department of Critical Care, University Hospital La Princesa, ISS La Princesa, Autonomous University of Madrid, Madrid, Spain; ^12^CIBERES, Carlos III Health Institute, Madrid, Spain; ^13^Department of Medicine, Surgery and Pharmacy, University of Sassari, Sassari, Italy

**Keywords:** extracorporeal CO_2_ removal, acute distress respiratory syndrome, asthma, chronic obstructive pulmonary disease, mechanical ventilation, continuous renal replacement therapy

## Abstract

Technological advancements have facilitated the application of extracorporeal-carbon-dioxide removal (ECCO_2_R) in managing acute respiratory-failure (ARF), including both hypoxemic and hypercapnic forms. A non-systematic literature review (PubMed, Medline, Embase, Google Scholar; January 2000–November 2024) identified randomized-controlled-trials (RCTs) and real-world evidence (RWE) on ECCO_2_R, alone or combined with continuous renal replacement therapy (CRRT). A multidisciplinary panel of intensivists, anesthesiologists, and nephrologists from Italy, Portugal, and Spain assessed clinical integration of ECCO_2_R. Key considerations included identifying ideal candidates, such as patients with acute respiratory distress syndrome (ARDS), chronic obstructive pulmonary disease (COPD), asthma exacerbations, alongside initiation timing and discontinuation criteria. For ARDS, recommended initiation thresholds included driving pressure ≥15 cm H_2_O, plateau pressure ≥28 cm H_2_O, pH < 7.28, and respiratory-rate >25 breaths/min. In COPD or asthma exacerbations at risk of non-invasive ventilation (NIV) failure, triggers included pH ≤ 7.25, RR ≥ 30 breaths/min, Intrinsic-PEEP ≥ 5 cm H_2_O, signs of respiratory fatigue, paradoxical abdominal motion, and severe distress. Absolute contraindications were uncontrolled bleeding, refractory hemodynamic instability, or lack of vascular access. Relative contraindications included moderate coagulopathy and limited access. The panel concluded ECCO_2_R may support selected adults with ARDS or obstructive lung disease, though further RCTs and high-quality prospective studies are needed to guide practice.

## Introduction

Acute respiratory failure (ARF), marked by impaired gas exchange, requires precise interventions (1). Mechanical ventilation (MV), while essential for supporting gas exchange, risks ventilator-induced lung injury (VILI) due to uneven lung distension, leading to systemic inflammation and organ failure (2–4). Extracorporeal CO_2_ removal (ECCO_2_R) has emerged as a promising strategy to manage inefficient CO_2_ elimination, facilitating lung-protective ventilation (LPV) with lower tidal volumes (VT) and airway pressures (5–13).

ECCO_2_R has diverse applications depending on clinical scenarios. In acute respiratory distress syndrome (ARDS), it reduces the intensity of invasive MV. In obstructive airway pathology, including chronic obstructive pulmonary disease (COPD) and asthma, as well as in lung transplantation (LTX) candidates, it decreases ventilatory workload, facilitates spontaneous breathing, and can prevent intubation (5–13). ECCO_2_R is particularly effective in managing hypercapnic acidosis, mitigating risks in ARDS and COPD patients and allowing an adjustment of the ventilatory parameters that minimizes lung injury. Compared to extracorporeal membrane oxygenation (ECMO), ECCO_2_R offers partial gas exchange support depending overall on CO_2_ decrease with almost no effect on oxygenation, with a much simpler implementation, making it suitable for intubated and non-intubated patients (14–20).

Studies have demonstrated that ECCO_2_R facilitated enhanced gas exchange and minimized VILI by allowing ultra-protective ventilation with reduced tidal volumes and airway pressures (21). During the COVID-19 crisis, Cambria et al. underscored its utility in stepping down ventilatory support, thereby improving the clinical management of critically ill patients (22). Consequently, ECCO_2_R has emerged as a pivotal adjunct in managing life-threatening respiratory failure, either as a bridge to recovery or as supportive intervention during pulmonary rehabilitation.

With ongoing technological progress, ECCO_2_R's clinical indications are broadening within intensive care settings due to its capacity to deliver vital respiratory support. However, updated guidelines from the European Society of Intensive Care Medicine caution against its routine use for non-COVID-19-related acute respiratory distress syndrome (ARDS), unless within the context of randomized controlled trials (23). This position stems from a lack of evidence showing mortality reduction. Similarly, for COVID-19-associated ARDS, the strong recommendation against routine use persists, though it is supported by moderate-certainty evidence due to indirect study designs (23).

In addition, the COVID-19 pandemic highlighted ECCO_2_R's value, especially when combined with continuous renal replacement therapy (CRRT) (24, 25). Pasero et al. (24) in an observational study, suggested that low-flow ECCO2R with CRRT significantly reduced driving pressures and intensive care unit (ICU) stay in moderate ARDS patients with acute kidney injury (AKI), though it did not impact 28-day mortality. Additionally, ECCO_2_R benefits patients with air leaks, improving hypercapnia and lung recovery (26).

Despite its advantages, ECCO_2_R can affect pulmonary and hemodynamic function and is associated with adverse events (AEs) involving the patient, the circuit, or mechanical failures (7, 10–13, 18, 27–30). Major AEs are frequently associated with veno-arterial cannulation, with risk factors influenced by the selection of vascular access and cannula characteristics (12–14, 18, 28–30). However, as the use of veno-arterial cannulation declines, single central venous cannulation with a double-lumen catheter has become the preferred approach. Documented complications of ECCO_2_R included membrane clotting, hemolysis, thrombocytopenia, significant bleeding, pump malfunction, catheter displacement, and infectious complications (12–14, 18, 28–30).

Additionally, anticoagulation with heparin, which was essential for maintaining ECCO_2_R performance, contributed to hemorrhagic complications (12–14, 18). However, the relatively high incidence of bleeding complications may be attributable to anticoagulation therapy and the elevated blood trauma associated with centrifugal pumps, which were originally engineered for high-flow systems (such as ECMO), particularly when operating with low blood volume processing (31). These findings underscore the need for advancements in blood pump technology to enhance safety and effectiveness at low flow rates.

This document aims to enhance the understanding and application of ECCO_2_R support, either alone or in combination with CRRT, in clinical practice, particularly for ARDS and acute COPD/asthma exacerbations. It synthesizes the latest evidence, establishes criteria for patient selection and intervention timing (encompassing initiation and weaning), and provides practical insights into ECCO_2_R's limitations and implementation, bridging gaps where formal guidelines may be lacking (32, 33).

## Methods

On September 16, 2024, a meeting was convened to evaluate the feasibility of conducting a comprehensive review on the use of ECCO_2_R across various clinical scenarios. The expert panel was composed of specialists in intensive care medicine, anesthesiology, and nephrology, chosen for their expertise and experience in mechanical ventilation and ECCO_2_R. The panel collaboratively selected and endorsed a set of key topics addressing the indications, strengths, and limitations of ECCO_2_R support in clinical practice.

Two separate face-to-face meetings were held to facilitate panel member interaction in their native languages: the first on October 3, 2024 for the Iberian Group, and the second on October 30, 2024 for the Italian Group. During these meetings, the panel engaged in extensive discussions on various practical aspects of ECCO_2_R use in routine clinical practice. Key topics included identifying the patient population most likely to benefit from ECCO_2_R support, determining the appropriate timing for initiating treatment (with a focus on clinical and gasometrical parameters), and defining the criteria for discontinuing ECCO_2_R support. These considerations were addressed both for ECCO_2_R used alone and in combination with CRRT.

Based on the information gathered during the two face-to-face meetings, an initial draft was prepared and subsequently reviewed during a virtual meeting held on November 27, 2024. After incorporating all the suggested revisions agreed upon by the panel members, the final document was reviewed and approved by the two study coordinators (PPT and FJPG). It was then circulated to all panel members for their final review and subsequent approval.

### Search strategy and eligibility criteria

A comprehensive but not systematic search of PubMed, Medline, Embase, and Google Scholar databases was conducted to identify randomized controlled trials (RCTs) and real-world evidence (RWE) studies evaluating the use of ECCO_2_R, either alone or in combination with CRRT, in various clinical conditions from January 2000 to March 31, 2025.

The search strategy employed Medical Subject Headings (MeSH) terms, including “Extracorporeal CO_2_ removal” OR “ECCO_2_R.” Additionally, a search was conducted using the MeSH terms “Extracorporeal CO_2_ removal” OR “ECCO_2_R” AND “Renal replacement therapy.”

To ensure a comprehensive and unbiased review of the literature on ECCO_2_R, a structured, multi-step search and selection strategy was employed. The initial search was supplemented by a manual screening of the reference lists from all included studies to identify additional relevant publications not retrieved through database queries. Furthermore, a free-text search of titles and abstracts was conducted using a range of clinically pertinent keywords, including “acute respiratory distress syndrome,” “chronic obstructive pulmonary disease,” “obstructive lung diseases,” “acute exacerbated chronic obstructive pulmonary disease,” “asthma,” and “respiratory dialysis.”

The selection process was performed in two stages. First, titles and abstracts were initially screened to exclude clearly irrelevant articles. Second, the full texts of potentially eligible studies were reviewed to confirm their inclusion based on predefined criteria. Studies were eligible if they evaluated the use of ECCO_2_R, either as a standalone support or in combination with CRRT, across various clinical scenarios. Exclusion criteria encompassed animal and *in vitro* studies, editorials, articles lacking clinical data applicable to human care, case reports, and case series with fewer than 10 participants. However, select case reports involving fewer than 10 patients were included when deemed to have significant relevance to the early development and clinical implementation of ECCO_2_R support. Additionally, only articles published in English, French, Portuguese, Italian, or Spanish were considered.

To minimize selection bias, two independent reviewers (FJPG and PT) conducted the study screening and selection. Discrepancies were resolved through discussion and consensus. This rigorous methodology was designed to provide a thorough and objective synthesis of current evidence regarding ECCO_2_R technologies and their clinical applications.

## Results

### Description of panel members characteristics

The panel members were expert clinicians who routinely administer ECCO_2_R support or support the renal specialist evaluation in patients who undergo CO_2_ removal treatment at various clinical centers across Italy, Portugal, and Spain.

The panel members had experience using different ECCO_2_R devices currently available in the European market at the time. All the panel members were familiar with different ECCO_2_R devices, with a median (interquartile range) experience of 4 (3.0–5.5) years.

#### Panel members centers protocols

Most panel members centers implement protocols for ECCO_2_R support (62.5%; 5/8), with variations influenced by regional preferences. Participant centers are guided by pathophysiological principles for lung protection and follow expert panel recommendations outlined in consensus guidelines (21).

The pathophysiological approach incorporated prone positioning for patients with an arterial partial pressure of oxygen to inspired oxygen fraction (PaO_2_/FiO_2_) ratio below 150 mmHg while excluding cases of refractory hypoxemia. CRRT initiation focuses on life-threatening acid-base disturbances, prescribing ≥25–30 mL/kg/h dialysate, without pre-dilution and achieving an effective blood flow (Qb) of 450 mL/min.

### Clinical applications of ECCO_2_R

#### Application of ECCO_2_R in ARDS

**Figure 1:**
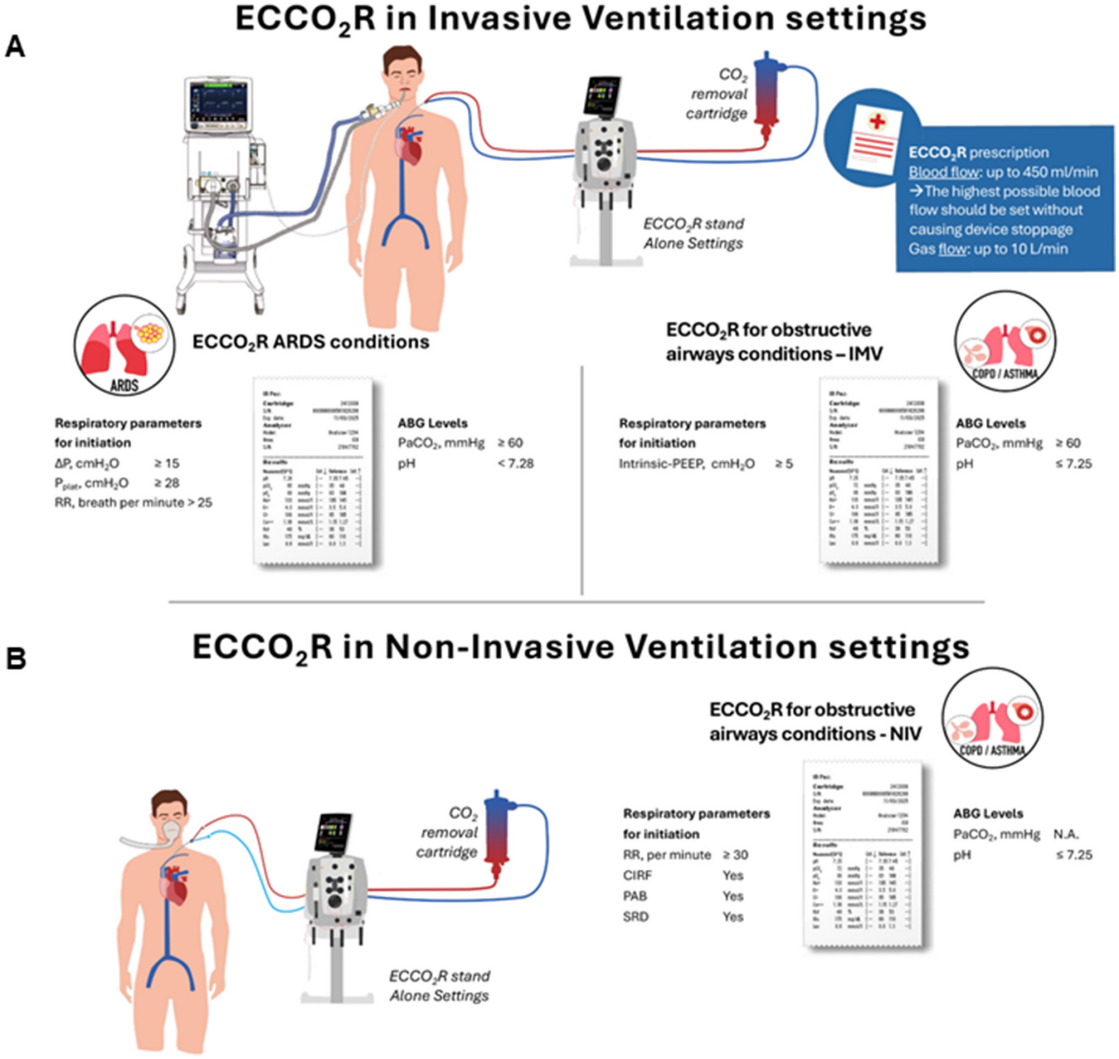
Applications and initiation parameters of extracorporeal CO_2_ removal in acute respiratory distress syndrome and obstructive airway diseases in invasive **(A)** and non-invasive **(B)** mechanical ventilation. Schematic representation of the clinical applications of ECCO_2_R in ARDS and obstructive airway diseases, under both invasive mechanical ventilation (IMV) and non-invasive ventilation (NIV) settings. The diagram details the respiratory parameters and arterial blood gas (ABG) thresholds for treatment initiation, along with the basic initiation parameters of ECCO_2_R. ABG, arterial blood gas; ARDS, acute respiratory distress syndrome; ECCO_2_R, extracorporeal CO_2_ removal; IMV, invasive mechanical ventilation; NIV, non-invasive ventilation; RR, respiratory rate; CIRF, clinical indicators of respiratory failure; PAB, paradoxical abdominal breathing; SRD, severity of respiratory dyspnea.

The primary respiratory parameters that, according to the expert panel, guide the decision to initiate or discontinue ECCO_2_R support in patients with ARDS, along with their respective cut-off values, are presented in Table 1 and Figure 1A.

**Table 1:** Criteria for initiating and discontinuing extracorporeal CO_2_ removal (ECCO_2_R) based on respiratory parameter thresholds in patients with moderate acute respiratory distress syndrome (ARDS).

**Respiratory parameter**	**Initiation^a^**	**Discontinuation^a, b^**
ΔP, cm H_2_O	≥15	< 14
P_plat_, cm H_2_O	≥28	< 28
PaCO_2_, mmHg	≥60	N.A.
pH	< 7.28^*^	>7.35
RR, per minute	>25	< 25

^*^In the context of respiratory acidosis following the optimization of ventilator settings. These data reflect the range established by the panel members.

^a^These parameters reflect the expert group's opinions, according to their clinical experience and specific practice setting. Nevertheless, they are consistent with those recently outlined in the 2022 European roundtable consensus (32).

^b^These parameters are applicable when conventional protective ventilation, utilizing a tidal volume of 6 ml/kg of predicted body weight, has already been established and with a Sweep Gas 0l/min test on ECCO_2_R device.

ΔP, driving pressure; Pplat, plateau pressure; PaCO_2_, arterial partial pressure of CO_2_; RR, respiratory rate; N.A., not applicable.

*Initiation and discontinuation of ECCO2R in supporting protective ventilation for ARDS patients:*

- Initiation of the support:
  ○ **Expert panel opinion:**
    ▪ Driving pressure (ΔP), Plateau pressure (Pplat), Arterial partial pressure of CO_2_ (PaCO_2_), pH, and respiratory rate (RR) have been identified as key respiratory parameters to evaluate when determining the initiation of ECCO_2_R in sedated patients with moderate ARDS receiving MV.
      - Patients with severe ARDS should be considered only during the early stages of the condition, provided they respond to treatment and show no signs of refractory hypoxemia.

- Discontinuation of the support:
  ○ **Expert panel opinion:**
    ▪ The key respiratory parameters for determining the discontinuation of ECCO_2_R in patients with moderate ARDS undergoing MV are ΔP, Pplat, PaCO_2_, pH, and RR.

##### The rational basis for utilizing ECCO_2_R in ARDS

ARDS is a life-threatening condition characterized by impaired gas exchange, resulting in oxygenation and/or CO_2_ elimination failure. With a mortality rate from 27 to 45%, ARDS usually requires invasive MV (9, 34–37). However, some patients continue to experience refractory hypoxia and/or hypercapnia despite optimal ventilation settings. The use of ECCO_2_R in ARDS is based on insights into VILI (4, 38, 39). The ARDSNet trial showed that reducing VT to 6 ml/kg decreased mortality (40) although 30% of patients still experienced pulmonary hyperinflation, indicating potential benefits from further VT reduction (2, 41). ΔP is the parameter most closely associated with mortality in patients with ARDS (42). It is closely related to respiratory system compliance (Crs) and depends on the set VT as well as the set PEEP (positive end expiratory pressure). Higher VT leads to higher ΔP, whereas higher PEEP also increases ΔP if it results in overdistension. Reducing ΔP has been shown to decrease pro-inflammatory factors in the BAL of patients with ARDS (2). Therefore, reducing the VT or ΔP in these patients is of particular interest when ΔP values exceed 15 cm H_2_O (39). However, reducing VT below 6 ml/kg can lead to severe hypercapnia and associated complications, which limits feasibility (43–46). Conversely, a *post-hoc* analysis of the SUPERNOVA trial found that ECCO_2_R devices with higher CO_2_ extraction capacity enabled more consistent reduction of tidal volume to 4 ml/kg in ARDS patients and were associated with fewer adverse events, such as hemolysis and bleeding, compared to lower-capacity systems (47).

ECCO_2_R has emerged as a promising solution to mitigate hypercapnia while enabling further reductions in VT, ΔP, and respiratory rate in ARDS patients. By facilitating CO_2_ clearance, ECCO_2_R supports ultra-low VT ventilation, reducing Pplat, ΔP, and mechanical power, all while maintaining clinically acceptable levels of PaCO_2_ and pH. This approach offers a viable strategy to mitigate VILI (21, 35, 48–51).

The SUPERNOVA study, a multicenter phase II trial involving 95 patients with moderate ARDS (PaO_2_/FiO_2_ 100–200 mmHg), demonstrated that ECCO_2_R could facilitate ultraprotective ventilation (VT 4 ml/kg, Pplat ≤ 25 cm H_2_O). By 8 and 24 h, 78% and 82% of patients achieved the desired ventilation settings, respectively (27). At day 28, 73% of patients were alive, and 62% were discharged alive. ECCO_2_R was effective in stabilizing pH levels and reducing PaCO_2_, but adverse events occurred in 39% of patients, including severe complications like brain hemorrhage and pneumothorax. The study also indicated that patients with higher alveolar dead space fraction (ADF) or lower respiratory system compliance (Crs) benefited most from ECCO_2_R treatment (27, 52). In the study conducted by Fanelli et al. (10), it was demonstrated that a reduction in ΔP of ~5 cm H_2_O was achieved when ECCO_2_R support was applied, without any observed changes in PaCO_2_ or pH in the arterial blood gas analysis.

More recently, the REST trial, however, found no significant reduction in 90-day mortality with ECCO_2_R combined with low VT ventilation compared to standard care, emphasizing the need for further investigation to clarify its efficacy in larger populations (11).

However, this study had several limitations: first, the inclusion criteria focused on oxygenation (PaO_2_/FiO_2_ < 150 mmHg), which is not the primary aim of ECCO_2_R. In fact, most patients did not meet ECCO_2_R criteria (injurious ventilation and severe acidosis). A secondary analysis of the trial showed a reduction in mortality in patients with a high ventilatory ratio, a parameter related to dead space and PaCO_2_, and thus more suitable candidates for ECCO_2_R (20). Second, the ECCO_2_R was delivered with a device using a centrifugal pump, which, unlike a roller pump, significantly decreases hydraulic efficiency when blood flow is below 1 L/min, leading to high shear stress, hemolysis, and platelet destruction (53). Third, the protocol involved a 15.5 Fr catheter and anticoagulation targeting an aPTT of 45–90 s, increasing hemorrhage risk. Finally, most centers lacked experience with the technique (11). A recent study by Monet et al. (54) further underscored the critical role of ECCO_2_R operational parameters, particularly blood pump speed, in determining the efficacy of the technique. The findings emphasized that, beyond the clinical indication, the precise adjustment of both ventilatory and ECCO_2_R settings was essential for optimizing therapeutic outcomes.

A meta-analysis encompassing 49 studies, including both observational studies and randomized controlled trials, examined adult ICU patients undergoing ECCO_2_R (51). The findings demonstrated that in patients with ARDS, ECCO_2_R led to a reduction in PaCO_2_ and an increase in arterial pH. Additionally, PaO_2_/FiO_2_ significantly improved, while Pplat and respiratory rate (RR) decreased. Notably, the reduction in VT reached statistical significance exclusively in ARDS patients (51).

While cohort studies confirm ECCO_2_R's ability to promptly reduce PaCO_2_ and correct acidosis, allowing lower RR, tidal volume, and plateau pressures (51), its predictive validity remains limited since randomized trials have not yet shown a mortality benefit and adverse events remain frequent (11).

In summary, ECCO_2_R is a promising tool for enhancing protective ventilation in moderate-severe conditions of ARDS patients, but evidence regarding outcomes of these patients remains inconclusive, with ongoing studies needed to better define its role and balance the potential benefits against risks such as acidosis and device-related complications (55).

When protective ventilation with low VT is insufficient to safeguard the lungs, and further reduction in VT would result in an unacceptable rise in CO_2_, leading to respiratory acidosis from alveolar hypoventilation, ECCO_2_R must be considered.

#### Application of ECCO_2_R in COPD/asthma exacerbations

The primary respiratory parameters that, according to the expert panel, guide the decision to initiate or discontinue ECCO_2_R support in patients with COPD/Asthma Exacerbations are outlined in the Table 2 and Figure 1A, along with their respective cut-off values.

**Table 2:** Criteria for initiating and discontinuing extracorporeal CO_2_ removal (ECCO_2_R) based on respiratory parameter thresholds in patients with obstructive airway pathology, including chronic obstructive pulmonary disease (COPD) and asthma exacerbations^**^.

**Respiratory parameter**	**COPD patients at risk of NIV failure**		**Intubated COPD patients** ^*^	
	**Initiation**	**Discontinuation^a^**	**Initiation**	**Discontinuation^a^**
pH	≤ 7.25	>7.25	≤ 7.25	>7.25
RR, *per minute*	≥30	< 30	N.A.	N.A.
CIRF	Yes	No	N.A.	N.A.
PAB	Yes	No	N.A.	N.A.
SRD	Yes	No	N.A.	N.A.
PaCO_2_, *mmHg*	N.A.	N.A.	≥60	< 60
Intrinsic-PEEP, *cmH_2_O*	N.A.	N.A.	≥5	< 5

^*^Including: intubated COPD patients, or those experiencing dynamic hyperinflation or impaired CO_2_ elimination.

^**^Patients experiencing acute exacerbation of asthma are also subject to these parameters. In such instances, the primary objective should be to minimize the respiratory rate to the greatest extent feasible. This approach, combined with the use of ECCO_2_R support to mitigate respiratory acidosis, is crucial for effectively reducing hyperinflation.

^a^These parameters are applicable when conventional protective ventilation, utilizing a tidal volume of 6 mL/kg of predicted body weight, has already been established and with a Sweep Gas 0l/min test on ECCO_2_R device.

COPD, chronic obstructive pulmonary disease; NIV, non-invasive ventilation; RR, respiratory rate; CIRF, clinical indicators of respiratory failure; PAB, paradoxical abdominal breathing; SRD, severity of respiratory dyspnea; PaCO_2_, Partial pressure of CO_2_; Intrinsic-PEEP, Auto-positive end-expiratory pressure.

Initiation and discontinuation of ECCO_2_R in supporting protective ventilation for COPD patients:

- Initiation of the support:
  ○ **Expert panel opinion:**
    ▪ *In COPD patients at risk of non-invasive ventilation (NIV) failure*, key respiratory parameters for assessing the need to initiate ECCO_2_R include arterial pH, RR, clinical indicators of respiratory failure, paradoxical abdominal breathing, and the severity of respiratory dyspnea (Table 2, Figure 1B).
    ▪ In *intubated COPD patients, or those experiencing dynamic hyperinflation or impaired CO*_2_ *elimination*, key respiratory parameters for evaluating the need to initiate ECCO_2_R include arterial pH, PaCO_2_ and intrinsic-PEEP.

In the context of hyperinflation, distinguishing between static and dynamic hyperinflation is crucial (56). Static hyperinflation arises from excessive PEEP settings, whereas dynamic hyperinflation, prevalent in COPD patients, results from expiratory flow limitation due to bronchial constriction, leading to air trapping (57). Insufficient exhalation time exacerbates this condition, increasing dead space and CO_2_ levels, perpetuating a vicious cycle (58). Intrinsic-PEEP, while informative, can be misleading as it depends on ventilator settings (57). ECCO_2_R effectively reduces CO_2_ levels, contributing to mitigate dynamic hyperinflation in patients on spontaneous ventilation, increasing expiratory time, and thereby supporting MV (56–58).

- Discontinuation of the support:
  ○ **Expert panel opinion:**
    ▪ In COPD patients at risk of NIV failure, key respiratory parameters for assessing the need to discontinue ECCO_2_R include arterial pH, RR, clinical indicators of respiratory failure, paradoxical abdominal breathing, and the severity of respiratory dyspnea.
    ▪ In intubated COPD patients, or those experiencing dynamic hyperinflation or impaired CO_2_ elimination, key respiratory parameters for assessing the need to discontinue ECCO_2_R include arterial pH, pCO_2_, and intrinsic-PEEP.

##### The rational basis for utilizing ECCO_2_R in obstructive airway pathology and weaning from MV

NIV remains the gold standard for managing acute hypercapnic respiratory failure, particularly in conditions like COPD or Asthma exacerbations (59). However, NIV fails in ~20%−30% of cases, leading to the need for intubation and invasive MV, which is associated with higher mortality rates compared to NIV alone. In these situations, combining ECCO_2_R support with NIV has emerged as a promising strategy to reduce NIV failure, prevent intubation, and improve patient outcomes (13, 18, 60).

ECCO_2_R enhances NIV efficacy by lowering respiratory metabolic load, reducing RR, increasing expiratory time and so reducing dynamic hyperinflation, and intrinsic PEEP. By avoiding invasive MV and intubation, ECCO_2_R could also minimize risks associated with analgo-sedation, such as hemodynamic instability, prolonged weaning, and neurological complications (13, 18). Moreover, ECCO_2_R reduces the work of breathing, decreasing CO_2_ production by respiratory muscles and PaCO_2_, thereby supporting earlier extubation or weaning from MV (14, 61).

In a multicenter and retrospective study, the use of a pumpless extracorporeal assist (PECLA) system in 21 COPD patients who failed NIV resulted in a 90% avoidance of intubation, along with a reduction in PaCO_2_ levels and improved pH. However, the study did not observe significant differences in mortality or ICU length of stay between the ECCO_2_R and control groups. The authors concluded that while ECCO_2_R effectively prevents intubation, its impact on survival remains inconclusive (62).

In addition, Azzi et al. (14) evaluated the effectiveness and safety of ECCO_2_R in patients with acute exacerbation of COPD experiencing NIV failure. According to the results of this study, ECCO_2_R improved pH and PaCO_2_, reduced intubation needs (85% avoided), and shortened ICU (18 vs. 30 days) and hospital length of stay (29 vs. 49 days) compared to the control group. Despite some bleeding complications, major complications were rare. ECCO_2_R showed lower ventilator-associated pneumonia rates and reduced 90-day mortality (15% vs. 28%), highlighting its potential as a safe and effective alternative to invasive MV (14).

Furthermore, Stommel et al. (51), in their systematic review and meta-analysis, demonstrated that in patients with COPD, ECCO_2_R significantly reduced PaCO_2_ and increased arterial pH. While the RR showed a statistically significant decrease, the PaO_2_/FiO_2_ ratio and VT did not exhibit significant changes.

Other studies have reported that in COPD patients with NIV failure, ECCO_2_R reduced intubation rates (26, 60, 63) and hospital mortality (60). However, some discrepancies and concerns have emerged from other studies, highlighting the need for careful patient selection and further research to clarify the safety profile of ECCO_2_R (64, 65).

A recent European expert consensus established key criteria for initiating ECCO_2_R support in COPD patients with acute exacerbations of COPD. Indications for ECCO_2_R initiation included the lack of PaCO_2_ reduction and failure to decrease RR while on NIV. The treatment goals for these patients were to improve patient comfort, maintain a pH above 7.30–7.35, reduce PaCO_2_ by 10–20%, decrease RR to 20–25 breaths/min, wean from NIV, decrease bicarbonate (HCO_3_-), and maintain hemodynamic stability (27, 66).

ECCO_2_R has also proven beneficial in facilitating weaning from invasive MV in patients with severe respiratory acidosis. In a retrospective analysis, Morelli et al. (63) highlighted the ability of ECCO_2_R to facilitate weaning from invasive MV, with six out of 12 patients being successfully weaned and surviving to discharge, while five were awake and breathing spontaneously during ECCO_2_R support.

In a study by Elliot et al. (67), the addition of pumpless ECCO_2_R allowed for successful weaning from invasive MV in two patients with severe acute asthma by correcting hypercapnia and acidosis. Abrams et al. (68) demonstrated that ECCO_2_R supported successful extubation in five COPD patients after only 24 h of invasive MV.

Finally, the VENT-AVOID trial, evaluated the impact of ECCO_2_R on ventilator-free days in patients with COPD exacerbations patients either failing NIV or struggling to wean from invasive MV (69). This study showed that ECCO_2_R exhibited a trend toward increased ventilator-free days in the invasive MV group, although without statistical significance, although did not improve ventilator-free days in the NIV group. However, this study has several limitations that should be taken into account. These include a small patient sample size, limited experience at the participating centers, unclear indications for ECCO_2_R use, the application of a centrifugal pump system, the use of a large-bore catheter (15.5 Fr), and the implementation of a permissive anticoagulation protocol (65)”.

In summary, based on the current evidence, ECCO_2_R represents a valuable intervention for managing acute hypercapnic respiratory failure in obstructive respiratory patterns, especially in COPD exacerbations, by improving the efficacy of NIV, preventing the need for invasive MV, and potentially facilitating earlier extubation in case of invasive MV. While ECCO_2_R support has demonstrated positive outcomes in reducing NIV failure and aiding weaning from MV, its safety and survival benefits remain uncertain. Observational studies suggest that ECCO_2_R can reduce complications and enhance patient recovery, but further randomized controlled trials are needed to clarify its role in clinical practice and refine patient selection criteria.

#### ECCO_2_R in combination with CRRT

Initiation and discontinuation of ECCO_2_R in combination with CRRT

- Indications to start CRRT in ECCO_2_R patients:
  ○ Potassium (K ≥ 6 mEq/L + electrocardiogram (ECG) anomalies not responsive to medical therapy.
  ○ pH anomalies (metabolic component in acidosis, pH < 7.10).
  ○ Hyperhydration not responsive to diuretics (very positive balance with diuresis reduction despite maximum diuretic administration).
  ○ KDIGO 2012 Guidelines (70):
    ▪ Start CRRT when life threatening anomalies in fluid, electrolyte and acid-base balance exist.
    ▪ Consider the broader clinical context, the presence of condition that can be modified with CRRT and the trends of laboratory tests, rather than simple Blood urea nitrogen (BUN) and creatinine thresholds done when making the decision to start CRRT.

- RRT prescription steps during ECCO_2_R:
  ○ Dose calculation:
    ▪ Patient weight x 25–30 ml/kg/h → aim: to reach effluent dose of 20–25 ml/kg/h at least, considering downtime (70).
    ▪ Keep Prescribed^*^ dose > administered dose → aim: to maintain effluent dose ≥ 20–25 ml/kg/h.
    ^*^Prescribed dose increase depends on downtime that is center-specific and due mainly to the time needed to bag changes and central venous catheter malfunctioning assessments.

○ Dose distribution:
○ Avoid predilution → aim: to avoid the reduction of the real dose administered (due to blood dilution).
  ▪ Dilution Factor (DF) = Plasma Flow Rate (ml/hr)/[Plasma Flow Rate (ml/hr) + Pre-Filter Replacement Fluid Rate (ml/hr) + Pre Blood-Pump PBP Fluid Rate (ml/hr)].
    - Where Plasma Flow Rate (ml/hr) = Blood Flow Rate (ml/min) × 60 (min/hr) × (1 – hematocrit [HCT]).

○ Suggested methods of CRRT
  ▪ Continuous veno-venous hemodialysis (CVVHD): no convection, no predilution. Effluent dose = dialysate + weight loss (net ultrafiltration).
  ▪ Continuous veno-venous hemodiafiltration (CVVHDF) just post dilution:
    - Pre-dilution, they must consider the dilution factor and correct the previously prescribed depurative dose.
    - Effluent dose = dialysate + post dilution + weight loss (net ultrafiltration).
    - Suggestion: to set up a post dilution with a filtration fraction (FF) ≤ 20%.
      ○ Filtration Factor (FF)= total ultrafiltration (UF) rate/plasma flow rate.
      ○ Considering PBP and predilution 0.
      ○ Not considering drop in weight in this calculation.

**Figure 2:**
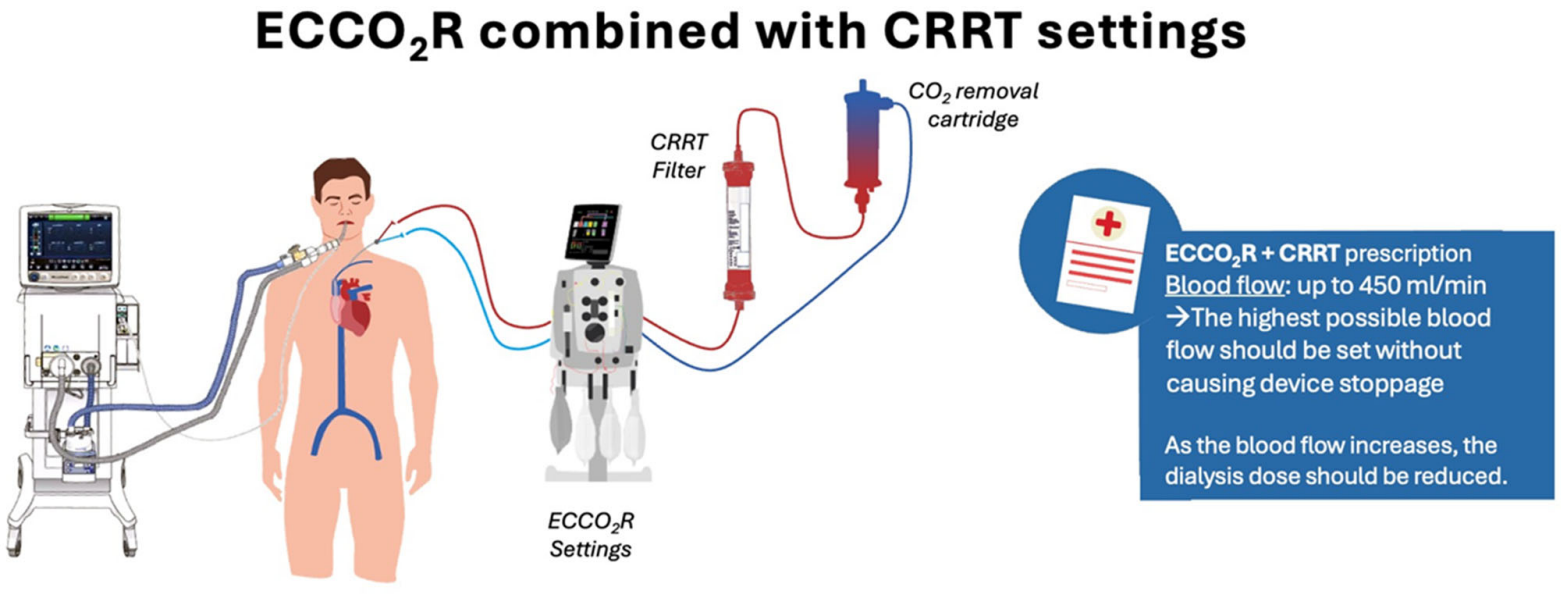
Applications and initiation parameters of extracorporeal CO_2_ removal in combination with continuous renal replacement therapies. Schematic representation of the clinical applications of ECCO_2_R in combination with CRRT. The process follows KDIGO 2012 AKI guidelines (See reference 70), emphasizing the importance of clinical context, fluid balance, and laboratory trends over isolated blood urea nitrogen and creatinine thresholds. Key considerations include dose calculation (25–30 mL/kg/h to achieve an effluent dose of ≥20–25 ml/kg/h), dose distribution (diffusive vs. post-dilution convective dose), and the impact of pre-dilution on effective clearance. Clinical insights highlight thresholds for initiating CRRT, the importance of maintaining prescribed vs. administered dose, and minimizing downtime. AKI, acute kidney injury; CRRT, continuous renal replacement therapy; ECCO_2_R, extracorporeal CO_2_ removal.

An overview of CRRT prescription steps during ECCO_2_R is shown in Figure 2.

##### The rational basis for utilizing ECCO_2_R in in combination with CRRT

Lung-kidney crosstalk refers to the bidirectional physiological and pathological interactions between these organs, crucial for maintaining homeostasis. The lungs regulate pH by managing CO_2_ levels, while the kidneys maintain acid-base balance through bicarbonate reabsorption and hydrogen ion excretion. Disruptions in one system often exacerbate dysfunction in the other (71, 72).

Pathological conditions, such as pulmonary-renal syndromes, fluid imbalances, and blood gas disturbances (e.g., hypoxemia, hypercapnia), illustrate this interplay. Pulmonary disorders can provoke systemic inflammation, worsening kidney injury, while renal dysfunction can hinder acid-base regulation, causing pulmonary complications (71, 73).

In critically ill patients, such as those with ARDS, lung-kidney interactions become increasingly significant. Pulmonary disorders lead to systemic inflammation, hypoxemia, and hypercapnia, impairing renal perfusion and function through venous congestion and hemodynamic changes (71, 74, 75). Conversely, kidney dysfunction exacerbates pulmonary complications by causing fluid overload, metabolic acidosis, and impaired gas exchange, further increasing respiratory effort (76, 77). MV compounds this interplay by reducing renal blood flow and contributing to oxidative stress and systemic mediator release (78).

ECCO_2_R can be implemented by integrating a membrane lung into standard RRT platforms, thereby enabling simultaneous extracorporeal respiratory and renal support (79, 80). Incorporating a hollow-fiber gas exchanger into a CRRT platform offers several practical benefits. These include technical simplicity and broader applicability, particularly in non-tertiary care settings, as the system typically does not require additional vascular access beyond that used for CRRT (12, 13).

Notably, the combination of ECCO_2_R with CRRT had been explored prior to the COVID-19 pandemic (5, 81–83). Nevertheless, this combined approach may be particularly beneficial in patients with COVID-19–associated ARDS, given several pathophysiological and clinical considerations. First, AKI is frequently observed in critically ill patients with COVID-19, with ~20% requiring RRT during their ICU stay (84). Second, mechanical ventilation has been identified as an independent predictor of mortality in individuals with AKI (85, 86). Third, the presence of elevated physiological dead space and reduced respiratory system compliance—hallmarks of COVID-19–related ARDS—may undermine the effectiveness of conventional lung-protective ventilation strategies (87–89).

The integration of ECCO_2_R with CRRT offers a promising solution for managing simultaneous respiratory and renal failure (79, 81, 90). This combined approach facilitates CO_2_ clearance and addresses metabolic derangements, improving outcomes in conditions such as hypercapnic acidosis and oliguric AKI (91–93).

This dual support shows potential to enhance clinical outcomes and reduce intensive care burdens, especially in patients with multi-organ dysfunction. While evidence supports its effectiveness, further research is necessary to optimize protocols and validate these findings in larger, more diverse populations (92).

### The role of anticoagulation in ECCO_2_R

Anticoagulation is essential in extracorporeal circuits to mitigate thromboembolic complications, especially in low-flow ECCO_2_R systems that are particularly prone to circuit clotting. Despite its critical role, no standardized anticoagulation protocol for ECCO_2_R has been universally established. Preventing system coagulation requires avoiding device stoppage caused by increased pre-pump pressure, which may result from suboptimal catheter positioning, patient hypovolemia, or excessively high blood flow rates. Therefore, blood flow should be maintained at the highest possible level without triggering device interruption.

Systemic anticoagulation with heparin is the most employed approach in both clinical trials and routine practice, using either unfractionated heparin (UFH) or low-molecular-weight heparin. However, this strategy carries risks such as bleeding and heparin-induced thrombocytopenia (94). In the absence of ECCO_2_R-specific randomized trials, anticoagulation management is largely extrapolated from veno-venous ECMO experience, where UFH remains the preferred anticoagulant. Under standard flow conditions, activated partial thromboplastin time (aPTT) targets typically range from 1.5 to 2.0 times baseline (~50–70 s). In low-flow ECCO_2_R (< 0.5 L/min), these targets are lowered to ~1.3 to 1.5 times baseline (40–55 s) to balance bleeding and thrombotic risks (95, 96).

Best practices include monitoring aPTT every 4–6 h after initiation or dose changes, daily platelet counts, and routine visual inspection of the circuit for clot formation. These ECMO-derived strategies are reasonable to apply in ECCO_2_R anticoagulation management (97–101). Table 3 summarizes anticoagulation strategies, aPTT targets, and monitoring recommendations adapted from existing ECMO evidence.

**Table 3:** Anticoagulation strategies for extracorporeal CO_2_ removal (ECCO_2_R): Adapted targets and monitoring guidance based on extracorporeal membrane oxygenation (ECMO) evidence.

**Element**	**Recommended approach**
Anticoagulant agent	Unfractionated heparin (UFH); alternative: direct thrombin inhibitors (DTIs) if UFH contraindicated (e.g., HIT) • ACT > 200 s: unchanged • ACT ≥ 180 ≤ 200 s increase infusion rate by 50% • ACT < 180 s: intravenous heparin bolus (12.5 IU/Kg) + increase infusion rate by 50%.
aPTT target range	Aim for **50–70 s**, equivalent to 1.5–2.5 × baseline; some centers may accept **40–60 s** in low-flow ECCO_2_R settings.
**Anti Xa target (if available)**	**0.3–0.5 IU/ml** (some centers may use lower range 0.2–0.3 IU/mL in low-flow circuits).
Monitoring frequency	aPTT every 2–4 h initially; anti Xa once daily or in first 24 h if used; ACT and viscoelastics (TEG/ROTEM) reserved for specific events or protocol use.
Use of ACT	Generally not preferred due to poor correlation with UFH levels; can supplement when point-of-care options available.
VET	Recommended early (first 24 h) to assess hemostatic profile; repeated as needed for bleeding or clot events.
Clinical context for ECCO_2_R	Given low-flow (< 0.5–1 L/min) nature of ECCO_2_R, aim for lower anticoagulation thresholds to limit bleeding risk while preventing circuit thrombosis.

ECCO_2_R, extracorporeal CO_2_ removal; ECMO, extracorporeal membrane oxygenation; UFH, unfractionated heparin; DTIs, direct thrombin inhibitors; HIT, heparin-induced thrombocytopenia; aPTT, activated partial thromboplastin time; ACT, activated clotting time; TEG, thromboelastography; ROTEM, rotational thromboelastometry; VET, viscoelastic testing.

**Rationale**

• UFH remains the standard anticoagulant for extracorporeal circuits given its reversibility and familiarity, but DTIs such as bivalirudin may be needed in heparin-induced thrombocytopenia scenarios (97–100).

• aPTT-based protocols (targeting 1.5–2.5 × baseline or 50–70 s) have been widely adopted, though associations between aPTT levels and bleeding/thrombotic events are inconsistent, particularly in the critical care setting (97–100).

• Anti-Xa assays are increasingly favored due to superior specificity for heparin activity and stability in the presence of confounding factors (e.g., low antithrombin, hemolysis) (97–100).

• ACT has limited utility and was poorly correlated with heparin dosing or clinical outcomes in ECMO patients (101).

• Viscoelastic assays (TEG/ROTEM) can provide real-time insight into coagulation dynamics and help guide component transfusion or adjustment of anticoagulation therapy (98, 100).

• In ECCO_2_R, the lower blood flow rates and reduced extracorporeal circuit surface suggest that moderately lower anticoagulation targets may be acceptable, balancing between bleeding and thrombosis.

#### Anticoagulation practices for ECCO_2_R:

- **Expert panel opinions:**
  ○ Universal agreement on anticoagulation:
    ▪ All panel members concur on the necessity of anticoagulation to ensure safe and effective ECCO_2_R implementation.
    ▪ Unfractionated heparin is universally used as the anticoagulant of choice in ECCO_2_R procedures.
    ▪ It is essential to prioritize appropriate anticoagulation practices to mitigate risks and safeguard patients undergoing ECCO_2_R support.

### Contraindications for ECCO_2_R: ensuring safe and optimal patient selection

The use of ECCO_2_R might be limited by several absolute and relative contraindications that must be carefully evaluated to ensure patient safety and treatment efficacy (3, 12, 13, 18, 30, 50, 51, 102). Almost all contraindications for ECCO2R are associated with anticoagulation.

Absolute contraindications include conditions with high risks of complications. They include active bleeding or the inability to establish vascular access is another absolute contraindication (3, 12, 13, 18, 30, 50, 51, 102).

Although hemodynamic instability that is refractory to medical therapy (i.e. severe shock or cardiovascular collapse) might be constituted an absolute contraindication for ECCO_2_R, as it may exacerbate hemodynamic stress, current evidence supports that if the instability is secondary to respiratory failure (i.e., failing right ventricle due to hyperinflation or severe respiratory acidosis), ECCO_2_R would probably be the solution. The hemodynamic benefits of ECCO_2_R in pulmonary hypertension secondary to hypercapnia and right ventricular (RV) dysfunction stem from its ability to modulate the pathophysiological cascade linking hypercapnia, respiratory acidosis, and pulmonary vasoconstriction. Hypercapnia-induced pulmonary vasoconstriction increases RV afterload, potentially exacerbating RV failure, particularly in patients with compromised cardiac reserve (18, 103–105). ECCO_2_R rapidly lowers PaCO_2_ and corrects acidosis, attenuating pulmonary vasoconstriction and reducing pulmonary arterial pressures and RV afterload (18, 103). This may improve cardiac output and stabilize hemodynamics when hypercapnia is the primary driver of RV strain. Furthermore, ECCO_2_R facilitates lung-protective ventilation strategies, potentially mitigating further RV compromise (18, 103).

Relative contraindications involve situations where risks are manageable but require cautious assessment. These include moderate to severe coagulopathy and limited vascular access. The presence of severe coagulopathy, such as an international normalized ratio (INR) > 2.5 or a platelet count < 30,000/μl, or instances of uncontrolled bleeding, poses significant challenges to the use of anticoagulation necessary for ECCO_2_R. While ECCO_2_R can technically be implemented without anticoagulation, these parameters necessitate a careful, individualized evaluation of its use. In such cases, it is imperative to operate without heparin, acknowledging the heightened risk of circuit failure due to recurrent clotting. This underscores the need for meticulous clinical judgment and consideration of patient-specific risks and benefits when deciding on ECCO_2_R in this context.

Other relative contraindications are inability to tolerate anticoagulation, or poor overall prognosis with advanced multi-organ failure, where ECCO_2_R is unlikely to provide meaningful benefit (3, 12, 13, 18, 30, 50, 51, 102).

The Table 4 outlines the critical absolute and relative contraindications for the use of ECCO_2_R, providing a comprehensive overview to guide safe and appropriate patient selection.

**Table 4:** Key contraindications for extracorporeal CO_2_ removal: a comprehensive overview of absolute and relative criteria.

**Type of contraindication**	**Specific contraindications**	**Opinion**
**Absolute**	Uncontrolled bleeding	Active bleeding precludes safe use of anticoagulation necessary for circuit patency^*^.
	Hemodynamic instability unresponsive to medical management	ECCO_2_R can exacerbate instability by requiring significant extracorporeal blood flows.
	Inability to cannulate	Mechanical barriers to vascular access preclude ECCO_2_R initiation.
**Relative**	Severe metabolic acidemia refractory to buffering strategies^**^	May indicate metabolic failure beyond the scope of ECCO_2_R support.
	Moderate to severe coagulopathy (e.g., INR > 2.5 or platelet count < 30,000/μl)	Increased bleeding risk necessitates careful anticoagulation adjustment and monitoring.
	Limited vascular access	Restricted access can complicate effective cannulation and circuit flows.
	Poor prognosis or advanced multi-organ failure	ECCO_2_R should not be used in patients where the burden of disease precludes meaningful benefit or survival.

^*^This contraindication applies not to the ECCO_2_R technique itself but to the requirement for patient anticoagulation. ECCO_2_R without anticoagulation requires individualized evaluation, balancing patient-specific risks and benefits while managing increased circuit failure risk from clotting.

^**^ECCO_2_R alone is not recommended in these cases. Instead, its individualized use combined with CRRT should be considered, as it may improve hemodynamics in severe shock with significant metabolic acidosis. ECCO_2_R is not intended to treat acidosis, but rather to address the underlying respiratory condition (e.g., ARDS, obstructive patterns) in the context of hemodynamic instability, if applicable.

ARDS, acute respiratory distress syndrome; CRRT, continuous renal replacement therapy; ECCO_2_R, extracorporeal CO_2_ removal; INR, international normalized ratio.

### Unmet needs in ECCO_2_R: key knowledge gaps and critical areas for future research

This paper reviewed the available evidence on the use of ECCO_2_R, both alone and in combination with CRRT, across various clinical settings, including ARDS and acute exacerbations of COPD and asthma with or without kidney failure. A critical analysis of the data revealed several key observations. First, all studies agreed that ECCO_2_R, whether standalone or integrated with CRRT, effectively managed hypercapnia and respiratory acidosis in mechanically ventilated patients. This was crucial, as regulating PaCO_2_ was essential for enabling lung-protective ventilation strategies (3, 12, 13, 18, 30, 50, 51, 102). Moreover, ECCO_2_R–CRRT allowed for CO_2_ removal with low blood flow, which improved clinical management and minimized adverse treatment effects (3, 12, 13, 18, 30, 50, 51, 102).

However, significant limitations existed. Notably, there was currently no evidence demonstrating that ECCO_2_R or ECCO_2_R–CRRT improved patient outcomes or reduced mortality (3, 12, 13, 18, 30, 50, 51, 94). This finding was common in studies involving critically ill patients, likely due to the complexity of the cases, small sample sizes, and short treatment durations. Furthermore, the studies were highly heterogeneous, involving diverse patient populations, outcomes, devices, and treatment parameters (12, 50, 51). A standardized ventilation protocol with predefined goals was often lacking, reducing the generalizability of the results (12, 50, 51).

Moreover, it would be advisable to emphasize the preferential use of roller pumps, as centrifugal pumps are not recommended for blood flows below 1–2 L/min due to their suboptimal performance at low flow rates (53). In ECCO_2_R therapies, both the pump type and the rate of blood flow are critical factors influencing the incidence of hemolysis. Evidence suggests that at higher flow rates, magnetically levitated pumps are associated with reduced hemolysis compared to conventional rotary pumps. However, under low-flow conditions (i.e., < 0.5 L/min), magnetically levitated pumps may paradoxically induce greater hemolysis than their rotary counterparts. Given that this review supports the application of low-flow ECCO_2_R modalities, the preferential use of peristaltic pumps over magnetically levitated systems may be advisable in such settings (53, 106).

In studies involving kidney failure, kidney outcomes and recovery were poorly reported, leaving the effectiveness of renal support provided by ECCO_2_R–CRRT treatment unclear (3, 12, 13, 18, 30, 50, 51, 102). Several other aspects remained underexplored, such as the optimal circuit configuration (e.g., positioning of the membrane oxygenator and hemofilter), the impact of dialysis buffers on systemic acid-base balance, and the management of anticoagulation (13, 107). These gaps highlighted the need for further research and caution in translating experimental findings into clinical practice.

## Study limitations

This review has several limitations that should be acknowledged. First, although a comprehensive search strategy was employed, including manual screening of reference lists and keyword-based free-text searches, there remains the possibility that relevant studies may have been missed, particularly unpublished data or articles indexed in databases not included in our search. Second, the review was limited to articles published in English, French, Portuguese, Italian, or Spanish, which may have introduced language bias and excluded relevant studies published in other languages. Third, case reports and small case series (fewer than 10 participants) were excluded to enhance the quality and generalizability of the findings; however, this may have led to the omission of potentially valuable insights, especially in rare or emerging clinical scenarios. Additionally, heterogeneity in study designs, patient populations, and outcome measures across the included studies may limit the ability to draw definitive conclusions regarding the efficacy and safety of ECCO_2_R in various clinical settings. Finally, as this is a narrative review and not a systematic review or meta-analysis, the level of evidence synthesis is inherently limited by the absence of quantitative data pooling.

## Conclusions

In conclusion, ECCO_2_R shows potential benefits for optimizing ventilatory strategies in respiratory failure patients. While it offers potentially relevant clinical advantages, its impact on patient prognosis, particularly in critically ill patients with multi-organ failure, requires further clarification.

The combination of ECCO_2_R and CRRT provides a flexible, cost-effective approach for patients with respiratory failure and kidney dysfunction. ECCO_2_R can be easily implemented in non-specialized centers using existing CRRT equipment, with appropriate training of the team in the technical handling of the device and the subsequent management of changes in ventilatory parameters that may benefit the patient. However, low-flow techniques may be insufficient for some patients, and ECMO should be considered for the most severely hypoxemic patients who do not respond to increasing PEEP.

Despite its effectiveness in managing hypercapnia and metabolic acidosis, the current ESICM guidelines (23, 32) recommend caution in using ECCO_2_R for ARDS outside of RCTs, stressing the need for further research.

Although evidence is limited, ECCO_2_R showed promising results, especially for hypercapnic respiratory failure, but additional well-designed trials are needed to fully assess its clinical impact. Given the challenges of large-scale trials in critically ill populations, ongoing clinical experience and cohort studies are essential for refining treatment protocols and identifying the most appropriate patient groups.
